# Supplementary material for: Stroboscopic light stimulation safety within and beyond laboratory settings: Observational evidence and practical guidance
Source: PLoS One. 2026 Oct 14;21(10):e0359958. doi: 10.1371/journal.pone.0359958 (PMC13667818; doi:10.1371/journal.pone.0359958)
Supplement: Peer review history

# Peer review: Decision Letter 0

**DOI:** 10.1371/journal.pone.0359958.r001

**Authors:** Gennady Cymbalyuk

22 Jul 2026

Dear Dr. Schwartzman,

Thank you for submitting your manuscript to PLOS ONE. After careful consideration, we feel that it has merit but does not fully meet PLOS ONE’s publication criteria as it currently stands. Therefore, we invite you to submit a revised version of the manuscript that addresses the points raised during the review process.

Before the manuscript can be considered for publication, the authors should address the remaining concerns regarding the clarity and organization of the manuscript, ensure that the literature review is current and adequately referenced, provide sufficient methodological detail for the included studies, more explicitly discuss the heterogeneity and limitations of the available evidence, and ensure that conclusions and recommendations are fully supported by the underlying data. In particular, the limitations associated with heterogeneous laboratory protocols, ad hoc reporting from commercial settings, the justification for age-related recommendations, and potential underestimation of adverse events should be clearly acknowledged and incorporated into the interpretation of the findings.

Please submit your revised manuscript by Sep 05 2026 11:59PM. If you will need more time than this to complete your revisions, please reply to this message or contact the journal office at plosone@plos.org. When you're ready to submit your revision, log on to https://www.editorialmanager.com/pone/ and select the 'Submissions Needing Revision' folder to locate your manuscript file.

- A letter that responds to each point raised by the academic editor and reviewer(s). You should upload this letter as a separate file labeled 'Response to Reviewers'.
- A marked-up copy of your manuscript that highlights changes made to the original version. You should upload this as a separate file labeled 'Revised Manuscript with Track Changes'.
- An unmarked version of your revised paper without tracked changes. You should upload this as a separate file labeled 'Manuscript'.

If you would like to make changes to your financial disclosure, please include your updated statement in your cover letter. Guidelines for resubmitting your figure files are available below the reviewer comments at the end of this letter.

If applicable, we recommend that you deposit your laboratory protocols in protocols.io to enhance the reproducibility of your results. Protocols.io assigns your protocol its own identifier (DOI) so that it can be cited independently in the future. For instructions see: https://journals.plos.org/plosone/s/submission-guidelines#loc-laboratory-protocols. Additionally, PLOS ONE offers an option for publishing peer-reviewed Lab Protocol articles, which describe protocols hosted on protocols.io. Read more information on sharing protocols at https://plos.org/protocols?utm_medium=editorial-email&utm_source=authorletters&utm_campaign=protocols.

As the corresponding author, your ORCID iD is verified in the submission system and will appear in the published article. PLOS supports the use of ORCID, and we encourage all coauthors to register for an ORCID iD and use it as well. Please encourage your coauthors to verify their ORCID iD within the submission system before final acceptance, as unverified ORCID iDs will not appear in the published article. *Only*  the individual author can complete the verification step; PLOS staff *cannot*  verify ORCID iDs on behalf of authors.

We look forward to receiving your revised manuscript.

Kind regards,

Gennady S. Cymbalyuk, Ph.D.

Academic Editor

PLOS One

Journal Requirements:

When submitting your revision, we need you to address these additional requirements.

1. Please ensure that your manuscript meets PLOS ONE’s style requirements, including those for file naming. The PLOS ONE style templates can be found at

https://journals.plos.org/plosone/s/file?id=wjVg/PLOSOne_formatting_sample_main_body.pdf and

https://journals.plos.org/plosone/s/file?id=ba62/PLOSOne_formatting_sample_title_authors_affiliations.pdf

2. Please provide additional details regarding participant consent. In the ethics statement in the Methods and online submission information, please ensure that you have specified (1) whether consent was informed and (2) what type you obtained (for instance, written or verbal, and if verbal, how it was documented and witnessed). If your study included minors, state whether you obtained consent from parents or guardians. If the need for consent was waived by the ethics committee, please include this information.

If you are reporting a retrospective study of medical records or archived samples, please ensure that you have discussed whether all data were fully anonymized before you accessed them and/or whether the IRB or ethics committee waived the requirement for informed consent. If patients provided informed written consent to have data from their medical records used in research, please include this information.

3. We note that the grant information you provided in the ‘Funding Information’ and ‘Financial Disclosure’ sections do not match.

When you resubmit, please ensure that you provide the correct grant numbers for the awards you received for your study in the ‘Funding Information’ section.

4. Thank you for stating the following financial disclosure:

"The author(s) received no specific funding for this work."

At this time, please address the following queries:

a)        Please clarify the sources of funding (financial or material support) for your study. List the grants or organizations that supported your study, including funding received from your institution.

b)        State what role the funders took in the study. If the funders had no role in your study, please state: “The funders had no role in study design, data collection and analysis, decision to publish, or preparation of the manuscript.”

c)        If any authors received a salary from any of your funders, please state which authors and which funders.

d)        If you did not receive any funding for this study, please state: “The authors received no specific funding for this work.”

Please include your amended statements within your cover letter; we will change the online submission form on your behalf.

5. Thank you for stating the following in the Competing Interests section:

"T.G. is affiliated with Lumenate Growth Ltd, G.L. is affiliated with Audyssey, and J.S. is affiliated with Roxiva Innovations Ltd. These affiliations are declared in the author list and are relevant to the commercial stroboscopic light stimulation contexts discussed in this article. All other authors declare no competing interests. This does not alter our adherence to PLOS ONE policies on sharing data and materials."

Please confirm that this does not alter your adherence to all PLOS ONE policies on sharing data and materials, by including the following statement: "This does not alter our adherence to  PLOS ONE policies on sharing data and materials.” (as detailed online in our guide for authors http://journals.plos.org/plosone/s/competing-interests).  If there are restrictions on sharing of data and/or materials, please state these. Please note that we cannot proceed with consideration of your article until this information has been declared.

Please include your updated Competing Interests statement in your cover letter; we will change the online submission form on your behalf.

6. Thank you for stating the following in the Competing Interests/Financial Disclosure * "T.G. is affiliated with Lumenate Growth Ltd, G.L. is affiliated with Audyssey, and J.S. is affiliated with Roxiva Innovations Ltd. These affiliations are declared in the author list and are relevant to the commercial stroboscopic light stimulation contexts discussed in this article. All other authors declare no competing interests. This does not alter our adherence to PLOS ONE policies on sharing data and materials.

We note that you received funding from a commercial source: "Lumenate Growth Ltd, Roxiva Innovations Ltd"

Please provide an amended Competing Interests Statement that explicitly states this commercial funder, along with any other relevant declarations relating to employment, consultancy, patents, products in development, marketed products, etc.

Within this Competing Interests Statement, please confirm that this does not alter your adherence to all PLOS ONE policies on sharing data and materials by including the following statement: "This does not alter our adherence to PLOS ONE policies on sharing data and materials.” (as detailed online in our guide for authors http://journals.plos.org/plosone/s/competing-interests).  If there are restrictions on sharing of data and/or materials, please state these. Please note that we cannot proceed with consideration of your article until this information has been declared.

Please include your amended Competing Interests Statement within your cover letter. We will change the online submission form on your behalf.

7. Your ethics statement should only appear in the Methods section of your manuscript. If your ethics statement is written in any section besides the Methods, please move it to the Methods section and delete it from any other section. Please ensure that your ethics statement is included in your manuscript, as the ethics statement entered into the online submission form will not be published alongside your manuscript.

8. If the reviewer comments include a recommendation to cite specific previously published works, please review and evaluate these publications to determine whether they are relevant and should be cited. There is no requirement to cite these works unless the editor has indicated otherwise.

[Note: HTML markup is below. Please do not edit.]

Reviewers' comments:

Reviewer’s Responses to Questions

**Comments to the Author**

1. Is the manuscript technically sound, and do the data support the conclusions?

Reviewer #1: Yes

Reviewer #2: No

**********

2. Has the statistical analysis been performed appropriately and rigorously?

Reviewer #1: Yes

Reviewer #2: No

**********

3. Have the authors made all data underlying the findings in their manuscript fully available?

The PLOS Data policy

Reviewer #1: Yes

Reviewer #2: Yes

**********

4. Is the manuscript presented in an intelligible fashion and written in standard English?

Reviewer #1: Yes

Reviewer #2: Yes

**********

Reviewer #1: This study provides a comprehensive, multi-center safety synthesis of stroboscopic light stimulation (SLS) across both controlled laboratory environments and large-scale commercial applications. While the absolute risk of severe adverse events are remarkably low, the expanding use of SLS beyond research necessitates standardized safety protocols. The authors developed the Sussex Strobe Safety Screening Questionnaire and provided actionable risk-mitigation guidelines to address this issue.

I have two minor feedbacks, first is regarding the upper age limit. Albeit status epilepticus is indeed more common over the age of sixty, this is mainly due to issues unrelated to photosensitivity, hence this upper limit may not be necessary in the context of this guideline.

Second issue is the ad-hoc nature of reporting in some settings, which may lead to underestimation of true side effects, therefore this should be addressed as a clear limitation.

Overall, it was a pleasure to review this paper. I wish the authors all the best.

Reviewer #2: Considering the expanded use of stroboscopic light stimulation (SLS) it is a good initiative to create a tool to detect at an early stage risks of getting epileptic seizures evoked by SLS. The paper first addresses all factors that might play a role in precipitating seizures followed by analyses of data on lab screening procedures and of commercial providers. The article is too long and describes many facets- it certainly needs some pruning with data from the commercial providers better be integrated in the introduction. Overall the article is difficult to read and information on photosensitivity is not up to date. Own experience with interviewing adolescents, who went to festivals, learned that it is considered normal that friends have epileptic seizures. Correctly, the authors state that the information from the commercial providers is not gathered in a scientifically sound way.

Some more comments:

- Photophobia, is relatively common in epilepsy , but true PSE is rare: this statement has no reference and it is unclear what true PSE means.

- The London 2012 promotional video was withdrawn because of reporting of seizures, not because of just concerns.

- Research lab data from several EU countries and Australia were used – more information is necessary about the type of study.

- A variety of lab based studies have been used introducing a lot of bias (different protocols and flash frequencies)- how can conclusions then being drawn?

- The conclusion that absolute risk of serious outcomes under closed eye SLS is low is not based on solid data.

- People above 55 yrs of age can still be photosensitive ( van Asch et al. Photosensitivity and self-induction in patients aged 50 and older. Epileptic Disord. 2024 Mar 18).

- Bright light is a common Migraine trigger : what is the reference for that?

**********

what does this mean?). If published, this will include your full peer review and any attached files.

If you choose “no”, your identity will remain anonymous but your review may still be made public.

**Do you want your identity to be public for this peer review?**  For information about this choice, including consent withdrawal, please see our Privacy Policy

Reviewer #1: No

Reviewer #2: **Yes:**  Dorothee Kasteleijn-Nolst Trenite

**********

[NOTE: If reviewer comments were submitted as an attachment file, they will be attached to this email and accessible via the submission site. Please log into your account, locate the manuscript record, and check for the action link "View Attachments". If this link does not appear, there are no attachment files.]

To ensure your figures meet our technical requirements, please review our figure guidelines: https://journals.plos.org/plosone/s/figures

You may also use PLOS’s free figure tool, NAAS, to help you prepare publication quality figures: https://journals.plos.org/plosone/s/figures#loc-tools-for-figure-preparation.

NAAS will assess whether your figures meet our technical requirements by comparing each figure against our figure specifications.

# Author comment: Author response to Decision Letter 1

**DOI:** 10.1371/journal.pone.0359958.r002

27 Aug 2026

Dear Editor and Reviewers,

We are grateful to the Editor and reviewers for their careful reading and constructive comments. We have revised the manuscript throughout. Most importantly, we now distinguish reported-event counts from estimates of incidence or prevalence, describe the retrospective and heterogeneous evidence more clearly, and have removed both the upper-age restriction and the unsupported claim that absolute risk is low. We have also expanded the Methods, added a study-level supplementary table, clarified what the 4SQ can and cannot establish, and updated the literature on photosensitivity, migraine, EEG screening, medication-associated seizure risk, and photobiological safety.

Reviewer comments appear in red, our responses in black, and revised manuscript text in blue. The same amendments are shown in bold in the marked manuscript. Page and line references below refer to the marked manuscript.

Academic Editor

Before the manuscript can be considered for publication, the authors should address the remaining concerns regarding the clarity and organization of the manuscript, ensure that the literature review is current and adequately referenced, provide sufficient methodological detail for the included studies, more explicitly discuss the heterogeneity and limitations of the available evidence, and ensure that conclusions and recommendations are fully supported by the underlying data. In particular, the limitations associated with heterogeneous laboratory protocols, ad hoc reporting from commercial settings, the justification for age-related recommendations, and potential underestimation of adverse events should be clearly acknowledged and incorporated into the interpretation of the findings.

Thank you for setting out the main concerns so clearly. We have reorganised and shortened the paper, expanded the Methods, and kept the laboratory and provider-reported evidence separate because it was collected in different ways. We have removed pooled estimates based on non-comparable sources and added a dedicated Limitations section.

We have also removed the upper-age restriction and updated the photosensitivity terminology. The revision now distinguishes photosensitive epilepsy, visually provoked seizures, a photoparoxysmal response (PPR), and photophobia. In particular, a PPR is an epileptiform EEG response to visual stimulation; it indicates photosensitivity but is not itself a clinical seizure or a diagnosis of epilepsy.

S1 Table now presents the available study-level information and marks information that was not reported or remains discrepant. The revised Limitations section states plainly which conclusions the data support and which they do not.

Changes made in Section 5.4, Limitations (page 20, lines 760–780):

This synthesis has several limitations. The contributors were selected as they had substantial experience delivering SLS; they were not sampled systematically, and reports were retrospective. Most laboratory studies involved young adult, predominantly university or student samples, apart from the broader depression studies; commercial user characteristics were less completely described. Participants were not counted more than once within an individual study, but some may have participated in more than one study because cross-study deduplication was unavailable. Study populations, devices, frequencies, brightness metrics, exposure durations, screening criteria, event definitions and follow-up procedures varied. This diversity helped identify safety considerations that recurred across delivery contexts, supporting guidance that is not tied to any single device or protocol. However, protocol heterogeneity, passive event reporting, non-comparable exposure denominators and the absence of verified links between reported events and individual users or sessions prevent pooled incidence estimation and comparisons of the relative safety of individual protocols. The provider-specific ratios should therefore be interpreted as descriptive reporting frequencies, not as incidence estimates or formal lower bounds. The absence of a reported event is not evidence that no event occurred. These limitations preclude conclusions about absolute risk, causal comparison of protocols or the effectiveness of screening. The 4SQ is a provisional checklist whose predictive performance requires formal evaluation.

Changes made in Section 1.3, Current screening practice (page 5, lines 191–196):

To address these gaps, we developed the Sussex Strobe Safety Screening Questionnaire (4SQ) as a provisional, evidence-informed, non-diagnostic checklist designed to support structured risk review and proportionate precautions. It integrates neurological, psychiatric, sensory, medical, substance, sleep and medication considerations, but its predictive performance and effect on adverse events will require formal evaluation.

Journal Requirements

1. PLOS ONE style and file naming

Please ensure that your manuscript meets PLOS ONE’s style requirements, including those for file naming.

We have checked the revised package against PLOS ONE style and will upload the following separately named files: PONE-D-26-19780_Revised_Manuscript.docx, PONE-D-26-19780_Marked_Manuscript.docx, PONE-D-26-19780_Response_to_Reviewers.docx, Fig1.tif, S1_Table.docx, S1_Text.docx, PONE-D-26-19780_Human_Participants_Research_Checklist.pdf, and PONE-D-26-19780_Revision_Cover_Letter.docx.

2. Participant consent

Please provide additional details regarding participant consent. In the ethics statement in the Methods and online submission information, please ensure that you have specified whether consent was informed and what type you obtained.

Thank you for asking us to clarify this. The present synthesis involved no new recruitment and no identifiable or individual-level participant data, so it did not require a separate participant-consent process. Participants in the contributing university studies provided informed consent under the relevant local ethics procedures; depending on the study format, consent was recorded in writing or electronically. Commercial providers supplied only aggregate, non-identifiable operational reports. We have made these distinctions explicit in the Methods and in the online submission information.

Changes made in the Methods, before Section 2.1 (pages 5–6, lines 200–214):

Ethics, consent and data governance

This study was a secondary analysis of anonymised, non-identifiable summary information supplied by contributing laboratories and commercial providers. We did not recruit participants for this synthesis and did not receive or access identifiable participant-level data. Under University of Sussex institutional guidance, analysis of these non-identifiable secondary data did not require new research ethics review or a separate participant-consent process. Participants in the contributing academic studies provided informed consent before taking part under the relevant local ethics procedures; depending on the study format, consent was recorded in writing or electronically. Commercial providers supplied aggregate, non-identifiable operational reports rather than participant-level research data. Their users were not recruited as research participants in this synthesis, and the providers did not seek research consent from them for this secondary analysis. Any service-use safety acknowledgements obtained by the providers were not treated as research consent.

3-4. Funding information and financial disclosure

We note that the grant information provided in the Funding Information and Financial Disclosure sections do not match. Please clarify all financial or material support, funder roles, and whether any authors received salaries from the funders.

Thank you for catching this mismatch. We have reconciled the Funding Information and Financial Disclosure entries and will use identical wording in Editorial Manager and the revision cover letter. The revised statement identifies each award and recipient, explains the role of the public funders and Collective Act Ltd, and makes clear that the commercial data providers did not fund this study. In line with PLOS ONE guidance, funding information is not repeated in the manuscript file.

Funding statement for the cover letter and Editorial Manager:

D.J.S. is supported by a Medical Research Council grant (UKRI083). A.K.S. is supported by European Research Council Advanced Investigator Grant 101019254. T.H. is supported by the Margaret Boden PhD Scholarship from the University of Sussex. F.M. was supported by the AHRC/UKRI Project SENSOR grant (AH/Y007638/1). J.W.S. is based at the NIHR University College London Hospitals Biomedical Research Centre and receives research support from the Marvin Weil Epilepsy Research Fund, the Academy of Medical Sciences and the NIHR.

F.M., T.H., D.J.S. and A.K.S. also received UK Government/UNBOXED 2022 funding, via Collective Act Ltd, to support their participation in the Dreamachine programme. The named public funders had no role in the analysis of this retrospective synthesis, the decision to publish, or preparation of the manuscript. UK Government/UNBOXED 2022 funding supported the Dreamachine programme from which part of the source data arose.

Collective Act Ltd delivered the Dreamachine programme and collected source data that contributed to this synthesis; its role in data collection and the involvement of the named authors are reported in the author-contribution and competing-interests statements. The commercial SLS providers supplied aggregate operational information but provided no financial support, equipment, or other material support for this study.

Related acknowledgement retained in the manuscript (page 20, lines 785–788):

The authors thank Collective Act Ltd and its Director, Jennifer Crook, for delivering the Dreamachine programme and collecting source data that contributed to this synthesis. The authors also thank Devraj Joshi (Whatever Together) and Cecelia Schwartzman for helpful discussions.

5-6. Competing interests and commercial support

Please provide an amended Competing Interests Statement that explicitly states the commercial funder, along with any other relevant declarations relating to employment, consultancy, patents, products in development, marketed products, etc., and confirm adherence to PLOS ONE policies on sharing data and materials.

Thank you for raising this point. The original online form incorrectly treated the commercial data providers as funders. They supplied aggregate operational data but no financial or material support for this study, so we have corrected the funding entry and expanded the competing-interests statement to describe the relevant commercial roles more explicitly.

The amended statement will appear in Editorial Manager and the cover letter, rather than in the manuscript file, in accordance with PLOS ONE guidance.

Amended competing-interests statement for the cover letter and Editorial Manager:

T.G. is a co-founder and director of Lumenate Growth Ltd. J.S. is a director of Roxiva Innovations Ltd. G.L. produces music under the name Audyssey and has collaborated with Roxiva. Lumenate and Roxiva market SLS products and supplied aggregate operational data for this study, but did not provide financial or material support. All other authors declare no competing interests. These relationships do not alter our adherence to PLOS ONE policies on sharing data and materials. The aggregate reported-event data, survey instrument, 4SQ and accompanying coding documentation are available at https://osf.io/nwkev/overview?view_only=c9979a478c734e7a9e46e50b5bed5c3a.

7. Placement of ethics statement

Your ethics statement should only appear in the Methods section of your manuscript.

As mentioned above, we have now consolidated the ethics, consent and data-governance statement within Section 2.0, Methods, and removed the duplicate end-matter statement. It now appears only in Section 2.0, Methods (pages 5–6, lines 200–214).

8. Suggested citations

If reviewer comments recommend specific publications, please evaluate whether they are relevant and should be cited.

Van Asch et al. (2024) is directly relevant to later-life photosensitivity and is now cited in Section 5.1. Fisher et al. (2022) supports the distinction between a PPR and a visually provoked seizure in Section 1.1 and the discussion of frequency-dependent provocation in Section 5.2. We also added references on migraine in Sections 1.1 and 5.1, EEG screening in Section 3.4, medication-associated seizure risk in Section 5.1 and S1 Text, and photobiological safety in Section 5.2. References added or newly relied upon in these revisions are listed at the end of this response in the same numerical order used in the manuscript.

Reviewer 1

1. Upper-age limit

Albeit status epilepticus is indeed more common over the age of sixty, this is mainly due to issues unrelated to photosensitivity, hence this upper limit may not be necessary in the context of this guideline.

Thank you for raising this point. We agree. The increased incidence and mortality of status epilepticus later in life do not, by themselves, imply greater photosensitivity, and so do not justify a photosensitivity-specific upper-age threshold. The 4SQ never excluded adults over 60; the upper-age restriction appeared only in the accompanying guidance, and we have now removed it. The key distinction is between safeguarding and clinical risk. Age 18 sets the lower boundary of this adult-use framework, while eligibility across adulthood depends on individual clinical history, not chronological age.

Changes made in Section 5.1, Age and safeguarding (pages 13–14, lines 503–514):

Rationale: Photosensitivity is most commonly identified in younger people, but it may persist or present after age 50 23,52. Although the incidence and mortality of status epilepticus increase in later life 53, this epidemiological pattern does not establish increased photosensitivity and therefore does not justify an upper-age threshold for SLS. Age alone should not be used as a contraindication to SLS.

Screening: Confirm that participants meet the study’s adult consent and safeguarding requirements. Assess neurological history, comorbidity, medication and capacity to communicate discomfort and stop exposure according to individual clinical context rather than chronological age.

Default decision: Retain age 18 as the lower boundary of this adult-use framework. Do not exclude or require clinical clearance solely on the basis of age; seek appropriate clinical input only when an individual’s history raises concern.

2. Ad hoc reporting and underestimation

The ad-hoc nature of reporting in some settings may lead to underestimation of true side effects, therefore this should be addressed as a clear limitation.

We agree that passive and ad hoc reporting may have led to under-ascertainment of events, particularly non-serious reactions, although the available data cannot show how many. We therefore now describe the commercial figures as provider-reported operational counts, have removed the combined 14/4.2-million rate, and state clearly that reporting was passive, the completeness of follow-up was unknown, and non-serious reactions were not captured systematically.

We have also clarified what the session totals represent. Provider A reported approximately one million users and 3.8 million sessions, so repeated use necessarily contributes to the session total. Provider B reported sessions rather than individual users, so repeat use may also be included in that total.

Changes made in Section 2.1, Commercial SLS providers (page 7, lines 255–270):

Data from two commercial SLS providers were obtained. Provider A operates a mobile application delivering closed-eye SLS through a smartphone flashlight, with frequency, brightness and duty cycle modulated in synchrony with audio. It reported approximately 3.8 million sessions from 2021 to 2025 and approximately one million users. As reported sessions substantially outnumber users, repeated use contributes to the session total. The dataset does not establish whether reported events were followed up independently or whether adverse-event capture was complete.

Provider B manufactures a configurable LED SLS device used in individual and group settings with more than 400,000 sessions over approximately five years. This is a minimum reported operational session count rather than a unique-user denominator: the same user could contribute more than one session, and additional consumer use was not included. Event reporting was passive and the completeness of follow-up is unknown. The two providers are described separately.

Further changes made in Section 5.4, Limitations (page 20, lines 760–780):

This synthesis has several limitations. The contributors were selected as they had substantial experience delivering SLS; they were not sampled systematically, and reports were retrospective. Most laboratory studies involved young adult, predominantly university or student samples, apart from the broader depression studies; commercial user characteristics were less completely described. Participants were not counted more than once within an individual study, but some may have participated in more than one study because cross-study deduplication was unavailable. Study populations, devices, frequencies, brightness metrics, exposure durations, screening criteria, event definitions and follow-up procedures varied. This diversity helped identify safety considerations that recurred across delivery contexts, supporting guidance that is not tied to any single device or protocol. However, protocol heterogeneity, passive event reporting, non-comparable exposure denominators and the absence of verified links between reported events and individual users or sessions prevent pooled incidence estimation and comparisons of the relative safety of individual protocols. The provider-specific ratios should therefore be interpreted as descriptive reporting frequencies, not as incidence estimates or formal lower bounds. The absence of a reported event is not evidence that no event occurred. These limitations preclude conclusions about absolute risk, causal comparison of protocols or the effectiveness of screening. The 4SQ is a provisional checklist whose predictive performance requires formal evaluation.

Reviewer 2

1. Length, organisation and integration of commercial evidence

The article is too long and describes many facets; it certainly needs some pruning, with data from the commercial providers better integrated in the introduction. Overall the article is difficult to read and information on photosensitivity is not up to date.

Thank you for this helpful criticism. We agree that the previous version of the manuscript tried to cover too much, which made the central argument harder to follow.

We have substantially shortened the main text, removed references not central to the revised scope, condensed the broader prevalence material, and removed the MRI comparison and pooled external estimates based on non-comparable outcomes and exposure contexts.

We also reorganised the evidential sequence. The Introduction now identifies the two commercial providers as a distinct source of operational evidence and states at the outset that the available data cannot establish population incidence or screening effectiveness. Because the laboratory and commercial information was collected in different ways, the Results describe these sources separately; the Discussion then brings them together only at the level of inference that the data support.

The Discussion now opens by explaining what these sources can and cannot establish. We also updated the photosensitivity literature, including Fisher et al. (2022) and van Asch et al. (2024), and now distinguish photosensitive epilepsy, visually provoked seizures, photoparoxysmal responses and photophobia.

Changes made in the Introduction, Section 1.0 (page 2, lines 75–82):

Against this backdrop, we synthesise cross-laboratory safety practice, retrospective reported-event data and operational reports from two commercial SLS providers, alongside a focused review of photosensitivity and sensory intolerance. Our aims are to describe current practice, identify recurrent tolerability and safety considerations, and develop a provisional, evidence-informed screening checklist for formal evaluation. The available data characterise reported adverse responses and recurrent safety considerations but cannot establish population incidence or screening effectiveness.

Changes made at the opening of the Discussion (page 13, lines 470–489):

Bringing these sources together provides a cross-setting account of reported adverse responses to SLS. Laboratory contributors reported 20 non-serious events and no seizures or syncope, whereas commercial providers separately reported nine and five medically important or otherwise notable events. Although these counts cannot establish incidence, absolute risk, causal attribution or screening effectiveness, they describe the events that were reported and identify what stronger evaluation requires: systematic surveillance, comparable exposure denominators and independent medical adjudication.

Building on this synthesis, we developed the 4SQ: a brief, plain-language, non-diagnostic checklist for structuring risk review and guiding proportionate precautions. It focuses on specific histories and sensitivities—including seizure history, previous visually provoked events, photophobia, migraine and sensory intolerance—rather than treating diagnostic labels as proxies for risk. Among the protocols and published guidance identified in this review, the 4SQ appears to be the most comprehensive publicly described SLS-specific pre-exposure checklist to date. What this establishes is breadth, not validity: it does not show that the 4SQ predicts adverse responses or reduces risk. These remain empirical questions. The next step is formal evaluation of its predictive performance, acceptability, and false-positive and false-negative rates.

2. Photophobia and 'true PSE'

Photophobia is relatively common in epilepsy, but true PSE is rare: this statement has no reference and it is unclear what true PSE means.

Thank you for catching this. “True PSE” was unclear, and the comparison with photophobia was not adequately referenced, so we removed it. We now distinguish photosensitive epilepsy, visually provoked seizures, photoparoxysmal responses (PPRs), and photophobia separately, citing Fisher et al. (2022) for visually sensitive seizures and PPRs and Digre and Brennan (2012) for photophobia.

Changes made in the Introduction, Section 1.1, Primary risk factor: photosensitive seizure (page 3, lines 89–99):

The main medical concern associated with SLS is a visually provoked seizure in an individual with photosensitivity. Photosensitive epilepsy (PSE) refers to epilepsy in which seizures can be provoked by visual stimuli. A photoparoxysmal response (PPR) is an epileptiform electroencephalographic response to visual stimulation; it indicates photosensitivity but is not itself a clinical seizure or an epilepsy diagnosis 23,24. Photosensitivity is more common in younger people 23 and in particular epilepsy syndromes 24, but can occasionally persist or present later in life 23,52. Photophobia instead refers to discomfort or intolerance associated with light and should not be treated as synonymous with PSE 35. These distinctions are important because the present synthesis includes medical events and non-serious tolerability outcomes.

3. London 2012 footage

The London 2012 promotional video was withdrawn because of reporting of seizures, not because of just concerns.

Thank you for correcting this. We agree that the footage was withdrawn following public reports of seizures rather than solely because of anticipatory concern. Because these reports did not medically verify those events, we have retained that distinction in the revised wording.

Changes made in the Introduction, Section 1.2 (page 5, lines 173–176):

The London 2012 Olympics’ promotional footage was withdrawn following public reports of seizures associated with the animation, although these reports did not medically verify these events 47.

4. Insufficient information about the laboratory studies

Research lab data from several EU countries and Australia were used; more information is necessary about the type of study.

This is a fair point. The earlier manuscript reported aggregate laboratory data without giving readers enough information about the individual studies.

We have expanded the Methods to describe the Qualtrics survey, the information requested from contributors, the event definitions, and the retrospective coding process. S1 Table now reports the available information at the most granular level supported by the source records. Most entries represent individual studies; where contributors supplied only portfolio-level information, the table says so rather than imposing an unsupported study-level split. It reports the purpose, population, setting, device, eye state, frequency or sequence, exposure, sessions, exposed denominator, screening, event ascertainment, reported events, follow-up, and unresolved source discrepancies. Where information was unavailable, we report it as NR (not reported in the available source records) rather than infer it. We also revised the laboratory overview to describe sample sizes as contributor-reported study totals rather than verified unique-person denominators.

Changes made in the Methods, Section 2.1 (page 6, lines 216–232):

On 24 July 2025, a structured Qualtrics portal was distributed to four SLS research groups and two commercial providers with substantial SLS experience; the data were accessed 28 days later, on 21 August 2025. It requested the contributor’s identity and reporting scope; reporting period; number and purpose of studies or sessions; device, eye state, frequency, brightness and duty cycle; participant or user counts; screening and risk-management procedures; and event narratives or supporting documents. A major event was defined as a seizure or another negative outcome with medical implications, and a non-serious event as a negative outcome that obstructed or stopped participation without reported medical implications. Contributors retrospectively determined which events met these definitions. Event descriptions were supplied by the contributing teams and were not independently confirmed against medical records. For online studies, follow-up was limited to information available to the original research teams. Narratives were grouped post hoc into descriptive symptom categories, using the primary stated reason for stopping when several symptoms were reported. Because populations, protocols, exposure units and surveillance procedures differed, we report sources separately and do not treat the synthesis as a meta-analysis.

Further changes made in Section 2.1, Research laboratories (page 6, lines 234–243):

Four research laboratories contributed data from studies conducted between 2020 and 2025 in the UK, Germany, the Netherlands and Australia. The studies involved mainly healthy participants, alongside adults reporting depressive symptoms. Contributor summaries described one study from Lab A (reported N = 20), twelve from Lab B (N = 461), six from Lab C (N = 480) and five from Lab D (N = 109). These are reported study totals rather than verified unique-person denominators: some study-level sample sizes were unavailable, and participation in more than one study could not be excluded. Where available, laboratories provided summary information on screening procedures and adverse-event logging.

New S1 Table (pages 1–3):

S1 Table. Available study- and portfolio-level characteristics of contributing laboratory datasets and adverse-event ascertainment

This table reports the most granular information available to the authors at revision. Some contributor records grouped multiple related studies or supplied only portfolio-level totals; these are identified rather than disaggregated without source confirmation. NR = not reported in the available source records. Values are descriptive, and unresolved discrepancies are stated explicitly. Event counts are reported events and should not be interpreted as incidence estimates.

5. Heterogeneous protocols and bias

A variety of lab based studies have been used introducing a lot of bias (different protocols and flash frequencies); how can conclusions then be drawn?

We agree that the heterogeneity rules out pooled incidence estimates and comparisons of the relative safety of different protocols. It does not, however, make the evidence uninformative. We have therefore narrowed the claims accordingly.

The key distinction is between description and inference. We use these data to describe the events that were reported and to identify safety considerations that recur across populations, devices, settings and frequencies. Patterns that recur across these different contexts can help structure a provisional, platform-agnostic checklist. They cannot establish causal risk factors, validate the 4SQ, demonstrate screening effectiveness or show that one protocol is safer than another.

For the same reason, we report the provider-specific event counts and session totals descriptively, not as incidence estimates or formal lower bounds. We do not pool results across sources or compare the safety of different protocols. The revised Limitations section makes these boundaries explicit.

Changes made in Section 5.4, Limitations (page 20, lines 760–780):

This synthesis has several limitations. The contributors were selected as they had substantial experience delivering SLS; they were not sampled systematically, and reports were retrospective. Most laboratory studies involved young adult, predominantly university or student samples, apart from the broader depression studies; commercial user characteristics were less completely described. Participants were not counted more than once within an individual study, but some may have participated in more than one study because cross-study deduplication was unavailable. Study populations, devices, frequencies, brightness metrics, exposure durations, screening criteria, event definitions and follow-up procedures varied. This diversity helped identify safety considerations that recurred across delivery contexts, supporting guidance that is not tied to any single device or protocol. However, protocol heterogeneity, passive event reporting, non-comparable exposure denominators and the absence of verified links between reported events and individual users or sessions prevent pooled incidence estimation and comparisons of the relative safety of individual protocols. The provider-specific ratios should therefore be interpreted as descriptive reporting frequencies, not as incidence estimates or formal lower bounds. The absence of a reported event is not evidence that no event occurred. These limitations preclude conclusions about absolute risk, causal comparison of protocols or the effectiveness of screening. The 4SQ is a provisional checklist whose predictive performance requires formal evaluation.

6. Unsupported conclusion that absolute risk is low

The conclusion that absolute risk of serious outcomes under closed eye SLS is low is not based on solid data.

We agree that our previous conclusion went beyond what these data support, and we have now removed the claim that absolute risk is low throughout the manuscript. We now distinguish explicitly between reported event counts and estimates of risk. The laboratory contributors reported 20 non-serious events, with no reported seizures or syncope, while the two commercial providers separately reported nine and five medically important or otherwise notable events. These are reported counts, not estimates of incidence or absolute risk. Commercial event capture was passive, non-serious reactions were not recorded systematically, and the completeness of case capture and follow-up is unknown; laboratory protocols, monitoring and follow-up were also heterogeneous. Events may therefore have been missed, but the extent of any under-ascertainment cannot be quantified. We now state explicitly that these data cannot establish incidence, absolute risk, causal attribution or screening effectiveness, and that the absence of a reported event is not evidence that no event occurred.

Changes made in the Abstract (pages 1–2, lines 37–48):

Because commercial adverse-event capture was passive and the laboratory protocols and monitoring procedures were heterogeneous, these figures cannot be used to estimate incidence or establish screening effectiveness. This synthesis informed a provisional Sussex Strobe Safety Screening Questionnaire (4SQ) and a layered, context-specific risk-management approach. The resulting framework distinguishes non-serious tolerability concerns from the principal medical risk of a visually provoked seizure in a susceptible individual, and supports conservative monitored familiarisation exposure, immediate stopping procedures and systematic adverse-event surveillance. The predictive performance of the 4SQ and the effectiveness of these precautions require formal evaluation; absolute SLS risk remains uncertain.

Changes made in the Introduction, Section 1.0 (page 2, lines 80–82):

The available data characterise reported adverse responses and recurrent safety considerations but cannot establish population incidence or screening effectiveness.

Changes made at the opening of the Discussion (page 13, lines 470–477):

Bringing these sources together provides a cross-setting account of reported adverse responses to SLS. Laboratory contributors reported 20 non-serious events and no seizures or syncope, whereas commercial providers separately reported nine and five medically important or otherwise notable events. Although these counts cannot establish incidence, absolute risk, causal attribution or screening effectiveness, they describe the events that were reported and identify what stronger evaluation requires: systematic surveillance, comparable exposure denominators and independent medical adjudication.

Changes made in Section 5.4, Limitations (page 20, lines 760–780):

See our response to the previous comment.

Changes made in the Conclusion (pages 20–21, lines 783–795):

This synthesis provides a cross-setting account of current SLS screening practice and reported adverse responses across heterogeneous laboratory and commercial settings. Laboratory contributors reported no seizures or syncope, whereas commercial providers separately reported nine and five medically important or otherwise notable events through passive channels. These observations describe reported events but cannot establish incidence, absolute risk or screening effectiveness. The 4SQ consolidates the recurrent safety considerations identified across these settings into a single, non-diagnostic framework for structured pre-exposure review, although its predictive performance remains to be established. Together, the synthesis and 4SQ provide a practical foundation for more consistent risk management and for formal evaluation of screening performance and adverse-event burden using harmonised definitions, unique-person and session denominators, and systematic follow-up.

7. Photosensitivity after age 55

People above 55 years of age can still be photosensitive (van Asch et al. Photosensitivity and self-induction in patients aged 50 and older. Epileptic Disord. 2024).

Thank you for drawing our attention to this study. We agree: older age does not rule out photosensitivity. Van Asch et al. (2024) identified photoparoxysmal responses (PPRs) in patients aged 50 years and older: these included photosensitivity persisting from childhood, PPRs associated with adult-onset epilepsy, and one incidental PPR. This retrospective clinical series does not establish population prevalence or the risk of visually provoked seizures in older adults. What it does show is that EEG photosensitivity can persist—or first be identified—later in life. We have therefore added the reference and removed the upper-age restriction from the accompanying guidance. The restriction applied to the guidance, not to the 4SQ questions themselves. Age 18 is retained only as the lower boundary for adult consent and safeguarding; older adults are not excluded solely on the basis of age, and decisions are based on individual clinical history, not chronological age.

Changes made in Section 5.1, Age and safeguarding (pages 13–14, lines 503–514):

Rationale: Photosensitivity is most commonly identified in younger people, but it may persist or present after age 50 23,52. Although the incidence and mortality of status epilepticus increase in later life 53, this epidemiological pattern does not establish increased photosensitivity and therefore does not justify an upper-age threshold for SLS. Age alone should not be used as a contraindication to SLS.

Screening: Confirm that participants meet the study’s adult consent and safeguarding requirements. Assess neurological history, comorbidity, medication and capacity to communicate discomfort and stop exposure according to individual clinical context rather than chronological age.

Default decision: Retain age 18 as the lower boundary of this adult-use framework. Do not exclude or require clinical clearance solely on the basis of age; seek appropriate clinical input only when an individual’s history raises concern.

8. Bright light and migraine

Bright light is a common migraine trigger: what is the reference for that?

Thank you for spotting the missing reference. We now cite Digre and Brennan (2012) and Schulte et al. (2015) and have qualified the wording. Bright or flickering light is often reported as a migraine trigger, but some apparent triggering may instead reflect photophobia during the premonitory phase. We therefore treat migraine and photophobia primarily as tolerability markers in this framework; the present data do not establish them as predictors of serious SLS events.

Changes made in Section 5.1, Migraine and photophobia (page 14, lines 530–541):

Migraine and photophobia

Rationale: Bright or flickering light is frequently reported as a migraine trigger, although some apparent triggering may reflect photophobia during the premonitory phase 35,38. Migraine, visual aura and photophobia are treated primarily as tolerability markers in this framework; the present data do not establish them as predictors of serious SLS events.

Screening: Assess migraine history, visual aura, photophobia and previous responses to bright or flashing light.

Default decision: Do not apply an automatic diagnosis-based exclusion. Defer or seek clinical advice where symptoms are active, severe or previously triggered by flashing light; otherwise consider lower intensity, shorter exposure and enhanced monitoring.

Additional Methodological Revisions

While addressing the reviewers’ comments, we found several related passages where the practical guidance needed clearer limits or stronger references. We revised these passages so that the recommendations remain useful without being presented as validated risk-reduction measures.

Frequency-dependent provocation

Changes made in Section 5.2, Practical risk mitigation (page 18, lines 700–709):

Clinically, photosensitivity is assessed using intermittent photic stimulation during EEG. Provocation depends jointly on flash frequency, luminance and contrast, colour, stimulated visual-field area, viewing distance, and exposure duration. It also depends on whether the eyes are open or closed and on the act of eye closure itself. Current review evidence identifies risk across approximately 3–60 Hz, with greatest sensitivity often around 15–20 Hz, rather than establishing a single universally safe upper-frequency threshold 24. SLS protocols should document and control the full stimulus profile, minimise exposure to more provocative combinations, use gradual monitored exposure where appropriate, and avoid describing sub-15-Hz stimulation as intrinsically safe.

Photobiological and ocular safety

Changes made in Section 5.2, Practical risk mitigation (pages 18–19, lines 711–721):

Photobiological safety cannot be inferred from luminous flux (lumens) alone because ocular hazard depends on spectral radiance or irradiance, source geometry, retinal image size and exposure duration. Closed-eye presentation should not be assumed to reduce seizure provocation: closing both eyes is not generally protective and can be more provocative in some photosensitive individuals 24. Neither blinking nor closed-eye presentation should be treated as a substitute for device-specific photobiological assessment. Protocols should measure relevant optical quantities at the participant position and document compliance with applicable guidance and standards, including the ICNIRP guidelines for incoherent visible and infrared radiation and IEC 62471 where relevant 56,57.

Status and intended function of the 4SQ

The earlier wording did not make the intended role of the 4SQ sufficiently clear. We now describe it directly as a non-diagnostic pre-exposure checklist for structured review of factors that may increase risk or reduce tolerability during SLS.

We also distinguish scope from validation. Among the protocols and guidance identified in our review, the 4SQ appears to be the most comprehensive publicly described SLS-specific pre-exposure checklist to date. That claim concerns what the checklist covers; its predictive performance and effect on adverse events still require formal evaluation.

Changes made in the Introduction, Section 1.3 (page 5, lines 191–196):

To address these gaps, we developed the Sussex Strobe Safety Screening Questionnaire (4SQ) as a provisional, evidence-informed, non-diagnostic checklist designed to support structured risk review and proportionate precautions. It integrates neurological, psychiatric, sensory, medical, substance, sleep and medication considerations, but its predictive performance and effect on adverse events will require formal evaluation.

Interpretation of a normal EEG

We placed this clarification in the Results section where the two studies using EEG pre-screening are described. It now states what a PPR and a normal EEG can, and cannot, tell us in this context.

Changes made in Section 3.4, EEG pre-screening (page 11, lines 399–408):

EEG pre-screening. In two studies, Laboratory D used a 10-minute EEG with intermittent photic stimulation for first-time SLS participants. Intermittent photic stimulation during EEG is an established clinical method for eliciting a PPR, but the incremental value of routine EEG screening in asymptomatic adults who have already undergone history-based screening is uncertain 51. A PPR may identify increased susceptibility, but a normal EEG does not establish that later exposure is risk-free and should not be treated as safety clearance. The present observational dataset did not compare screening strategies and cannot establish whether EEG screening, questionnaire screening or graded exposure is more effective.

Purpose of familiarisation exposure

We have kept familiarisation exposure in the guidance but narrowed the claim. It lets participants experience the stimulus under conservative conditions, practise stopping it, and notice immediate discomfort before the full session. It does not show that a participant is medically safe to proceed or guarantee tolerance of a longer, brighter, or otherwise different exposure.

Changes made in Section 5.2, Practical risk mitigation (page 18, lines 677–687):

SLS risk management should combine appropriate participant information, context-specific screening, controlled exposure, a readily available stop procedure, trained staff and systematic event documentation. Common practices across the contributors included briefing, an explicit opt-out and a brief supervised familiarisation exposure. This familiarisation allows participants to experience the stimulus at conservative intensity, practise the stop procedure and identify immediate discomfort before the full session. It is retained as a tolerability measure intended to reduce avoidable discomfort and early distress. The present data did not compare this practice experimentally, and a tolerated familiarisation exposure does not constitute medical clearance or guarantee tolerance of longer, brighter or otherwise different stimulation.

Medication-list disclaimer

We have retained the medication list because it can help researchers and organisers recognise medication-related contexts—including recent dose changes and withdrawal—that may affect seizure susceptibility. The revised text makes the boundary explicit: the list is an awareness aid, not prescribing guidance or a universal exclusion list.

Changes made in Section 5.1, Medication and withdrawal contexts (page 17, lines 636–651):

Medication and withdrawal contexts

Rationale: Medication-associated seizure risk depends on the drug, dose and context and may be concentrated during toxicity, rapid dose change, withdrawal, interacting illness or combinations of pro-convulsant factors 58,59. The supplementary medication list is therefore provided as an awareness aid for researchers and organisers, not as prescribing guidance or a universal exclusion list.

Screening: Record current medication, recent initiation or dose change, withdrawal and any history of medication-associated seizures. Where necessary, eligibility decisions should be informed by a clinician or pharmacist. Participants should not discontinue or alter prescribed medication in order to take part.

Default decision: Defer participation or obtain appropriate clinical advice when there is a specific high-risk medication, toxicity, withdrawal, recent dose change or relevant seizure history. Stable medication use should be assessed in relation to the study population, protocol and clinical oversight rather than treated as an automatic exclusion.

Corresponding disclaimer in S1 Text (page 1):

Interpretive note. This list is an awareness aid for researchers and organisers, not prescribing guidance or a universal exclusion list. Medication-associated seizure risk depends on the drug, dose and context. Eligibility decisions should therefore be specific to the protocol and, where necessary, informed by a clinician or pharmacist. Participants should not discontinue or alter prescribed medication in order to take part. See supplementary references 1,2.

References

23. Quirk JA, Fish DR, Smith SJM, Sander JWAS, Shorvon SD, Allen PJ. Incidence of photosensitive epilepsy: a prospective national study. Electroencephalogr Clin Neurophysiol. 1995;95(4):260-267. doi:10.1016/0013-4694(95)00118-I

24. Fisher RS, Acharya JN, Baumer FM, et al. Visually sensitive seizures: An updated review by the Epilepsy Foundation. Epilepsia. 2022;63(4):739-768. doi:10.1111/epi.17175

35. Digre KB, Brennan KC. Shedding Light on Photophobia. J Neuroophthalmol. 2012;32(1):68-81. doi:10.1097/WNO.0b013e3182474548

37. Steiner TJ, Stovner LJ. Global epidemiology of migraine and its implications for public health and health policy. Nat Rev Neurol. 2023;19:109–117. doi:10.1038/s41582-022-00763-1

38. Schulte LH, Jürgens TP, May A. Photo-, osmo- and phonophobia in the premonitory phase of migraine: mistaking symptoms for triggers? J Headache Pain. 2015;16(1):14. doi:10.1186/s10194-015-0495-7

47. Glendinning L. Please look away ... it’s the 2012 logo. The Guardian. https://www.theguardian.com/media/2007/jun/06/marketingandpr.olympics2012. June 6, 2007. Accessed October 13, 2025.

51. de Pémille CV, Rekik S, Amiel H, et al. Contribution of intermittent photic stimulation to routine EEG. Neurophysiol Clin. 2021;51(6):549-553. doi:10.1016/j.neucli.2021.10.003

52. van Asch CJJ, Spetgens WPJ, Bourez-Swart MD, et al. Photosensitivity and self-induction in patients aged 50 and older. Epileptic Disord. 2024;26(3):293-301. doi:10.1002/epd2.20209

53. Ong CT, Sheu SM, Tsai CF, Wong YS, Chen SC. Age-dependent sex difference of the incidence and mortality of status epilepticus: a twelve year nationwide population-based cohort study in Taiwan. PLoS One. 2015;10(3):e0122350. doi:10.1371/journal.pone.0122350

56. International Commission on Non-Ionizing Radiation Protection (ICNIRP). ICNIRP Guidelines on Limits of Exposure to Incoherent Visible and Infrared Radiation. Health Phys. 2013;105(1):74-96. doi:10.1097/HP.0b013e318289a611

57. International Electrotechnical Commission. IEC 62471:2006: Photobiological safety of lamps and lamp systems. Geneva: IEC; 2006.

58. Hitchings AW. Drugs that lower the seizure threshold. Adverse Drug Reaction Bulletin. 2016;298(1):1151-1154. doi:10.1097/FAD.0000000000000016

59. Ruffmann C, Bogliun G, Beghi E. Epileptogenic drugs: a systematic review. Expert Rev Neurother. 2006;6(4):575-589. doi:10.1586/14737175.6.4.575

Attachment Submitted filename: PONE-D-26-19780_Response_to_Reviewers.docx

# Peer review: Decision Letter 1

**DOI:** 10.1371/journal.pone.0359958.r003

**Authors:** Gennady Cymbalyuk

22 Sep 2026

Stroboscopic Light Stimulation Safety Within and Beyond Laboratory Settings: Observational Evidence and Practical Guidance

PONE-D-26-19780R1

Dear Dr. Schwartzman,

We’re pleased to inform you that your manuscript has been judged scientifically suitable for publication and will be formally accepted for publication once it meets all outstanding technical requirements.

Within one week, you’ll receive an e-mail detailing the required amendments. When these have been addressed, you’ll receive a formal acceptance letter and your manuscript will be scheduled for publication.

An invoice will be generated when your article is formally accepted. Please note, if your institution has a publishing partnership with PLOS and your article meets the relevant criteria, all or part of your publication costs will be covered. Please make sure your user information is up-to-date by logging into Editorial Manager at Editorial Manager® and clicking the ‘Update My Information' link at the top of the page. For questions related to billing, please contact billing support.

If your institution or institutions have a press office, please notify them about your upcoming paper to help maximize its impact. If they’ll be preparing press materials, please inform our press team as soon as possible -- no later than 48 hours after receiving the formal acceptance. Your manuscript will remain under strict press embargo until 2 pm Eastern Time on the date of publication. For more information, please contact onepress@plos.org.

Kind regards,

Gennady S. Cymbalyuk, Ph.D.

Academic Editor

PLOS One

Additional Editor Comments (optional):

I have reviewed your responses to Reviewer 1 and 2 and the corresponding revisions to the manuscript. Although the Reviewer 1 report on the revision 1 has not been submitted, I find that the reviewers' concerns have been adequately addressed.

Reviewers' comments:

Reviewer’s Responses to Questions

**Comments to the Author**

Reviewer #2: All comments have been addressed

**********

2. Is the manuscript technically sound, and do the data support the conclusions?

Reviewer #2: Yes

**********

3. Has the statistical analysis been performed appropriately and rigorously?

Reviewer #2: N/A

**********

4. Have the authors made all data underlying the findings in their manuscript fully available?

The PLOS Data policy

Reviewer #2: Yes

**********

5. Is the manuscript presented in an intelligible fashion and written in standard English?

Reviewer #2: Yes

**********

Reviewer #2: The authors have changed the manuscript substantially: it is now interesting to read and gives more clear information on how and why. Thank you for the answers and revisions made.

It is ready for publication

**********

what does this mean?). If published, this will include your full peer review and any attached files.

If you choose “no”, your identity will remain anonymous but your review may still be made public.

**Do you want your identity to be public for this peer review?**  For information about this choice, including consent withdrawal, please see our Privacy Policy

Reviewer #2: **Yes:**  Dorothee Kasteleijn-Nolst Trenite

**********

# Editor report: Acceptance letter

**DOI:** 10.1371/journal.pone.0359958.r004

**Authors:** Gennady Cymbalyuk

PONE-D-26-19780R1

PLOS One

Dear Dr. Schwartzman,

I'm pleased to inform you that your manuscript has been deemed suitable for publication in PLOS One. Congratulations! Your manuscript is now being handed over to our production team.

At this stage, our production department will prepare your paper for publication. This includes ensuring the following:

* All references, tables, and figures are properly cited

* All relevant supporting information is included in the manuscript submission,

* There are no issues that prevent the paper from being properly typeset

You will receive further instructions from the production team, including instructions on how to review your proof when it is ready. Please keep in mind that we are working through a large volume of accepted articles, so please give us a few days to review your paper and let you know the next and final steps.

Lastly, if your institution or institutions have a press office, please let them know about your upcoming paper now to help maximize its impact. If they'll be preparing press materials, please inform our press team within the next 48 hours. Your manuscript will remain under strict press embargo until 2 pm Eastern Time on the date of publication. For more information, please contact onepress@plos.org.

You will receive an invoice from PLOS for your publication fee after your manuscript has reached the completed accept phase. If you receive an email requesting payment before acceptance or for any other service, this may be a phishing scheme. Learn how to identify phishing emails and protect your accounts at https://explore.plos.org/phishing.

If we can help with anything else, please email us at customercare@plos.org.

Thank you for submitting your work to PLOS One and supporting open access.

Kind regards,

PLOS One Editorial Office Staff

on behalf of

Dr. Gennady S. Cymbalyuk

Academic Editor

PLOS One
